# Prevalence and correlates of psychological distress among 13–14 year old adolescent girls in North Karnataka, South India: a cross-sectional study

**DOI:** 10.1186/s12889-018-6355-z

**Published:** 2019-01-10

**Authors:** Tara S. Beattie, Ravi Prakash, April Mazzuca, Leslie Kelly, Prakash Javalkar, T. Raghavendra, Satyanarayana Ramanaik, Martine Collumbien, Stephen Moses, Lori Heise, Shajy Isac, Charlotte Watts

**Affiliations:** 1 Department of Global Health and Development, London School of Hygiene and Tropical Medicine, London, UK; 2 Karnataka Health Promotion Trust, Bangalore, Karnataka India; 3 School of Population and Public Health, University of British Columbia, Vancouver, Canada; 4 Departments of Community Health Sciences and Medicine and Medical Microbiology, University of Manitoba, Winnipeg, Canada; 5 Department of Population, Family and Reproductive Health, Bloomberg School of Public Health, Johns Hopkins University, Baltimore, MD USA

**Keywords:** Adolescent girls, School drop out, Child marriage, Sexual abuse, Psychological distress, Suicide ideation

## Abstract

**Background:**

Mental health disorders among adolescents have emerged as a major public health issue in many low and middle-income countries, including India. There is a paucity of research on the determinants of psychological distress, particularly among the poorest girls in the poorest communities. The purpose of this study was to assess the prevalence and correlates of different indicators of psychological distress among 13–14 year old low caste girls in rural, south India.

**Methods:**

Cross-sectional survey of 1191 low caste girls in two districts in north Karnataka, conducted as part of a cluster randomised-control trial. Bivariate and multivariate logistic regression analysis assessed correlates of different indicators of psychological distress.

**Results:**

More than one third of girls (35.1%) reported having no hope for the future. 6.9% reported feeling down, depressed or hopeless in the past 2 weeks. 2.1% reported thinking they would be better off dead or of hurting themselves in some way in the past 2 weeks. 1.6% reported sexual abuse, 8.0% rrecent eve teasing and 6.3% having no parental emotional support. Suicidal ideation was independently associated with sexual abuse (AOR 11.9 (3.0–47.0)) and a lack of parental emotional support (AOR 0.2 (0.1–0.5)). Feeling down, depressed or hopeless was independently associated with recent eve-teasing (AOR 2.9 (1.6–5.4)), a harassing or abusive school environment (AOR 3.9 (1.8–8.2)), being frequently absent (AOR 2.8 (1.5–5.5)) or having dropped out of school (AOR 2.1 (1.0–4.3)), and living in Vijayapura district (AOR 2.5 (1.6–4.1)). Having no hope for the future was independently associated with a range of factors, including recent “eve-teasing” (AOR 1.5 (1.0–2.4)), being engaged (AOR 2.9 (0.9–9.7)), not participating in groups (AOR 0.5 (0.4–0.6)) and a lack of emotional support (AOR 0.6 (0.4–0.7)).

**Conclusions:**

Rather than being a time of optimism, a third of low caste girls in rural north, Karnataka have limited hope for the future, with some contemplating suicide. As well as having important development benefits, interventions that address the upstream structural and gender-norms based determinants of poor mental health, and provide adolescent services for girls who require treatment and support, should have important benefits for girls’ psychological wellbeing.

**Trial registration:**

Prospectively registered at ClinicalTrials.GovNCT01996241. November 27, 2013

**Electronic supplementary material:**

The online version of this article (10.1186/s12889-018-6355-z) contains supplementary material, which is available to authorized users.

## Background

India has the largest adolescent population in the world, with 20% of the world’s adolescents [1]. The transition to adulthood, particularly for girls in rural settings, can start in early adolescence (10–14 years), with child marriage and school drop-out occurring soon after menarche. Early adolescence (10–14 years), is biologically dominated by puberty and the effects of the pubertal hormones on body morphology, and sexual and brain development [2]. Premature autonomy with early disengagement from parents and school can predict poorer health and wellbeing [3].

Mental health disorders among adolescents have emerged as a major public health issue in many low and middle-income countries (LMIC), including India. Common mental health disorders, such as depression and anxiety, often emerge during adolescence, with many persisting across the life course [4, 5]. After the onset of puberty, the risk of depressive disorders increases substantially, especially among girls, who are 1.5–2 times more likely than boys to be diagnosed with depression; this notable gender gap persists throughout the life-course [5]. Rather than being a static organ, the brain is known to be in a highly dynamic stage of development during adolescence, but the causes of common mental disorders in youth have been less researched. The heritability of mood disorders does not exceed 40%, suggesting a key role for social (e.g. gender roles) and environmental factors (e.g. hormonal changes associated with puberty) in explaining individual differences [5]. Global mortality data suggests suicide has surpassed maternal mortality as the leading cause of death among girls aged 15–19 years [6], although reliable statistics from LMIC are uncommon [7]. Suicidal ideation refers to thoughts of harming or killing oneself [8] and suicidal behaviours, including ideation and attempt, are a precursor for completed suicide [9, 10]. Loneliness and lack of parental support are associated with suicidal ideation among youth in South-East Asia and Western Pacific countries [11], and sexual abuse in childhood is associated with suicidal behaviours in adulthood [12].

Psychosocial wellbeing, which includes a lack of stress and shame, and believing one’s actions can affect one’s life, is a concept being developed in the economic and development literature, particularly in relation to the effects of cash transfer programmes [13, 14]. It moves away from the more medicalised definitions of mental health and its treatment, and examines the role of poverty and vulnerability on psychological wellbeing. A qualitative study of women in rural Maharashtra, India, found that mental illness was understood to be the product of cultural and socio-economic stressors [15], the most common being conflict with husbands and mothers-in-law, and poverty. Conversely, a small number of qualitative studies focusing on the social effects of cash transfer programmes have found that cash transfer recipients report considerable relief from the ‘worries of daily existence’ (anxiety and stress), increased dignity and pride, and increased control over their lives [14].

Gender refers to the distinguishing features of men and women that are socially constructed [16]. It influences the control that men and women have over the determinants of their health, including their economic position, social status and access to resources [17]. Examples of gender disadvantage among adolescents in the Indian context are the experience of violence (including “eve-teasing”), being married and bearing children during adolescence, diminished autonomy in decision making, increased burden of household chores and responsibilities, and absenteeism and drop-out from education. ‘Eve teasing’ of adolescent girls in public spaces (sexual harassment by men and adolescent boys) is commonly described in Indian settings. Although the victims may ostensibly not experience serious harm (name calling, whistling, being pelted with small stones), the importance of being seen to maintain a girl’s ‘sexual purity’ until she marries to protect the family’s honour means the fear of ‘eve teasing’, can lead to a family terminating a girl’s education and rushed early marriage [18]. Child marriage (marriage < 18 years) affects ~ 40,000 girl brides every day, with one in three marriages taking place in India [19]. Girl brides face higher rates of unintended pregnancy, early pregnancy, maternal morbidity and mortality, neonatal morbidity and mortality, and domestic violence than their unmarried peers [20–22]. For some brides, especially girls who are very young, ‘guana’ (marriage consummation) and cohabitation with their spouse can occur sometime after marriage, sometimes several years later. When brides move to their spouse’s household, which may be in a different village or district, they are usually removed from educational opportunities and have reduced/minimal contact with their family and peer network [23, 24]. They tend to have limited social networks, restricted social mobility, little autonomy, and little access to media and health messages [19, 25]. School drop-out affects 60 million early adolescents (aged 12–14) globally [26], with gender, poverty, marital status and rural location key risk factors [27, 28]. School drop-out reflects a process (rather than a single event) and is usually preceded by frequent absenteeism [29–32]. Leaving school restricts girl’s opportunities, including the level and quality of future employment [27], and is associated with increased child mortality and fertility [29]. There is a paucity of research from LMIC examining associations between child marriage or school dropout, and adolescent mental health and well-being.

The aim of the current research was to estimate the prevalence and determine the correlates for psychological distress among low caste (Scheduled Caste / Scheduled Tribe (SC/ST)) adolescent girls in rural, south India. We undertook secondary data analysis of baseline survey data conducted with 13–14 year old girls in 2014, as part of a cluster randomised control trial (RCT), evaluating an intervention called *‘Samata’*. Samata aims to reduce child marriage, delay the age at which girls start selling sex, and increase secondary school entry and retention, as a means to reducing HIV-related risk, among adolescent girls from the lower castes (scheduled caste/ scheduled tribe) living in two districts in north Karnataka [33].

## Methods

### Setting

Vijayapura and Bagalkote districts in northern Karnataka are poor rural areas where the predominant work is seasonal, agricultural labour. The scheduled caste and scheduled tribe people comprise approximately 20% of the population, [34] with most households (> 85%) living below the poverty line [35]. In this region, the *Devadasi* tradition of sex work is commonly practiced, in which adolescent girls from *Devadasi* families are dedicated to Gods and Goddesses and subsequently inducted into selling sex [36]. HIV rates among women who sell sex in this area were previously among the highest in India (~ 30%) [37].

### Participants

All SC/ST girls in the final year of primary school, living in 80 villages, were recruited in two cohort waves, one academic year apart. The questionnaire (Additional file 1), administered to the second cohort wave during their first term of secondary school (Sept-Dec 2014), included questions on psychological distress and it is this data that were used for this study.

### Measurements

The questionnaire comprised three parts: a brief demographic and economic questionnaire asked to an adult family member; a structured questionnaire asked by a female interviewer to adolescent girls in private in their own homes; and an anonymous short self-completed questionnaire containing sensitive questions (menarche, sexual abuse, eve-teasing, suicidal ideation) that girls completed by themselves (or in the case of low literacy levels, with the assistance of interviewers). Questionnaires were translated into Kannada and pre-tested before the survey.

#### Socioeconomic factors (Table 1)

Information on household sociodemographic characteristics (caste, orphan hood, household composition, literacy status, and household assets) were collected from an adult family member. Data were collected from adolescent girls (face-to-face interview) on her age, and her own and her parents’ migration for work.

**Table 1:** Associations of socioeconomic factors with psychological distress among adolescent girls (aged 13–14 years) in north Karnataka, south India, 2014

	% of the sample (*N* = 1191)	Do not have hope for the future			Feeling down, depressed or hopeless (past 2 weeks)			Suicidal thoughts (past 2 weeks)		
		Prevalence (*N* = 1177)	Univariate OR (95% CI)	*P* value	Prevalence (*N* = 1189)	Univariate OR (95% CI)	*P* value	Prevalence (*N* = 1169)	Univariate OR (95% CI)	*P* value
*Total*		413/1177 (35.1)			82/1189 (6.9)			25/1144 (2.1)		
Socioeconomic factors										
*Age (years)*										
≤ 13 years	96.6	401/1136 (35.3)	1.0		77/1148 (6.7)	1.0		22/1128 (2.0)	1.0	
14+ years	3.4	12/41 (29.3)	0.8 (0.4-1.5)	0.4	5/41 (12.2)	1.9 (0.7-5.1)	0.2	3/41 (7.3)	4.0 (1.1-13.8)	0.031
Median (years)	13.0									
*Caste*										
Scheduled caste	77.3	314/910 (34.5)	1.0		707/919 (7.6)	1.0		21/910 (2.3)	1.0	
Scheduled tribe	22.7	99/267 (37.1)	1.1 (0.8–1.5)	0.4	12/270 (4.4)	0.6 (0.3–1.1)	0.074	4/259 (1.5)	0.7 (0.2–2.0)	0.5
*Orphan hood*										
Both parents alive	84.1	334/988 (33.8)	1.0		72/1000 (7.2)	1.0		21/984 (2.1)	1.0	
One/both parents dead	16.0	79/189 (41.8)	1.4 (1.0–1.9)	0.035	10/189 (5.3)	0.7 (0.4–1.4)	0.3	4/185 (2.2)	1.0 (0.3–3.0)	1.0
*Number of siblings*										
0–2	36.6	158/432 (36.6)	1.0		31/436 (7.1)	1.0		5/427 (1.2)	1.0	
3	32.2	136/381 (35.7)	1.0 (0.7–1.3)	0.8	24/383 (6.3)	0.9 (0.5–1.5)	0.6	9/376 (2.4)	2.1 (0.7–6.2)	0.2
4–9	31.2	119/364 (32.7)	0.8 (0.6–1.1)	0.3	27/370 (7.3)	1.0 (0.6–1.8)	0.9	11/365 (3.0)	2.6 (0.9–7.6)	0.076
Median	3.0									
*Literacy household head*										
Illiterate	63.3	281/745 (37.7)	1.0		46/753 (6.1)	1.0		12/740 (1.6)	1.0	
Literate	36.7	132/431 (30.6)	0.7 (0.6–0.9)	0.014	36/435 (8.3)	1.4 (0.9–2.2)	0.2	13/428 (3.0)	1.9 (0.9–4.2)	0.1
*Household wealth index*										
Poorest	29.6	105/346 (30.4)	1.0		25/352 (7.1)	1.0		8/344 (2.3)	1.0	
Medium	33.4	158/394 (40.1)	1.5 (1.1–2.1)	0.006	22/398 (5.5)	0.8 (0.4–1.4)	0.4	9/392 (2.3)	1.0 (0.4–2.6)	1.0
Richest	37.0	150/437 (34.3)	1.2 (0.9–1.6)	0.24	35/439 (8.0)	1.1 (0.7–1.9)	0.6	8/433 (1.9)	0.8 (0.3–2.1)	0.6
*Parents migrated for work past year*^a^										
No	83.3	348/979 (35.6)	1.0		68/987 (6.9)	1.0		21/967 (2.2)	1.0	
Yes	16.7	63/193 (32.6)	0.9 (0.6–1.2)	0.4	13/198 (6.6)	0.9 (0.5–1.8)	0.9	4/196 (2.0)	0.9 (0.3–2.8)	1.0
*Girl migrated for work past year*										
No	91.6	383/1077 (35.6)	1.0		77/1089 (7.1)	1.0		21/1071 (2.0)	1.0	
Yes	8.4	30/100 (30.)	0.8 (0.5–1.2)	0.3	5/100 (5.0)	0.7 (0.3–1.8)	0.4	4/98 (4.1)	2.1 (0.7–6.3)	0.2
*District*										
Bagalkote	57.2	265/679 (39.0)	1.0		30/681 (4.4)	1.0		14/663 (2.1)	1.0	
Vijayapura	42.8	148/498 (29.7)	0.7 (0.5–0.8)	0.001	52/508 (10.2)	2.5 (1.6–3.9)	< 0.001	11/506 (2.2)	1.0 (0.5–2.3)	0.9

^a^Data missing for 6 participants (0.5%)

#### Gender disadvantage factors (Table 2)

In the face-to-face interviews, girls were asked about their marital and school-going status. Frequent absenteeism was defined as absent from school for ≥4 days in the past month. We defined her current (or last) school as having a harassing or abusive school environment if she responded yes to any of the following: sexual harassment of girls by other students; sexual harassment of girls by teachers/staff; harsh physical punishment by teachers; bullying by other students. Menarche, sexual abuse and eve- teasing experience were asked in the self-completed questionnaire. Sexual abuse was defined if anyone had ever (i) touched or fondled you in a sexual way when you did not want them to, (ii) tried to have sexual intercourse with you when you did not want them to, or (iii) forced you to have sex with them. Recent eve teasing (yes/no) was defined if she reported being sexually harassed or teased in the past 12 months (Sometimes as girl’s bodies mature, they begin to attract unwanted attention from boys and men. In the past 12 months, have you been sexually harassed or teased?), or being sexually harassed or teased at school, on the way to school or somewhere else in the village in the past 3 months (Have you been sexually teased or harassed… at school (Y/N)), on way to school (Y/N), somewhere else in the village (Y/N)) in the last 3 months),.

**Table 2:** Associations of gender disadvantage and psychosocial buffers with psychological distress among adolescent girls (aged 13–14 years) in north Karnataka, south India, 2014

	% of the sample (*N* = 1191)	Do not have hope for the future			Feeling down, depressed or hopeless past 2 weeks			Suicidal thoughts past 2 weeks		
		Prevalence (*N* = 1177)	Univariate OR (95% CI)	*P* value	Prevalence (*N* = 1189)	Univariate OR (95% CI)	*P* value	Prevalence (*N* = 1169)	Univariate OR (95% CI)	*P* value
*Total*		413/1177 (35.1)			82/1189 (6.9)			25/1144 (2.1)		
Gender disadvantage										
*Sexual abuse ever*^a^										
No	98.4	384/1103 (34.8)	1.0		66/1115 (5.9)	1.0		18/1102 (1.6)	1.0	
Yes	1.6	4/18 (22.2)	0.5 (0.2–1.6)	0.3	1/18 (5.6)	0.9 (0.1–7.1)	0.9	3/18 (16.7)	12.0 (3.2–45.3)	< 0.001
*Recent eve teasing*^*2*^										
No	92.0	369/1076 (34.3)	1.0		67/1087 (6.2)	1.0		21/1076 (2.0)	1.0	
Yes	8.0	43/94 (45.7)	1.6 (1.1–2.5)	0.027	15/95 (15.8)	2.9 (1.6–5.2)	0.001	4/92 (4.4)	2.3 (0.8–6.8)	0.14
*Harassing/abusive school environment*										
No	95.9	395/1128 (35.0)	1.0		71/1140 (6.2)	1.0		23/1121 (2.1)	1.0	
Yes	4.1	18/49 (36.7)	1.1 (0.6–2.0)	0.8	11/49 (22.5)	4.4 (2.1–8.9)	< 0.001	2/48 (4.2)	2.1 (0.5–9.1)	0.3
*Started Menstruating*^*3*^										
No	38.5	179/448 (40.0)	1.0	0.007	27/454 (6.0)	1.0	0.3	6/449 (1.3)	1.0	0.13
Yes	61.5	232/721 (32.2)	0.7 (0.6–0.9)		55/727 (7.6)	1.3 (0.8–2.1)		19/718 (2.7)	2.0 (0.8–5.1)	
*Marital status*										
Never married	95.3	389/1121 (34.7)	1.0		79/1134 (7.0)	1.0		23/1113 (2.1)	1.0	
Engaged	1.1	8/13 (61.5)	3.0 (1.0–9.3)	0.055	1/13 (7.7)	1.1 (0.1–8.7)	0.9	0/13 (0.0)	–	–
Married	3.6	16/43 (37.2)	1.1 (0.6–2.1)	0.7	2.42 (4.8)	0.7 (0.2–2.8)	0.6	2/43 (4.7)	2.3 (0.5–10.1)	0.3
*Schooling status*										
In school	84.6	342/996 (34.3)	1.0		58/1007 (5.8)	1.0		17/988 (1.7)	1.0	
Frequently absent	7.4	30/87 (34.5)	1.0 (0.6–1.6)	1.0	14/88 (15.9)	3.1 (1.6–5.8)	< 0.001	2/86 (2.3)	1.4 (0.3–6.0)	0.7
Dropped out	8.0	41/94 (43.6)	1.5 (1.0–2.3)	0.073	10/94 (10.6)	1.9 (1.0–4.0)	0.065	6/95 (6.3)	3.9 (1.5–10.0)	0.006
Psychosocial resources										
*Social Integration*										
*Membership and participation in informal groups*										
None/Low	32.1	180/377 (47.8)	1.0		28/380 (7.4)	1.0		10/370 (2.7)	1.0	
Medium/High	67.9	233/800 (29.1)	0.4 (0.3–0.6)	< 0.001	54/809 (6.7)	0.9 (0.6–1.4)	0.7	15/799 (1.9)	0.7 (0.3–1.5)	0.4
*New friends past year*										
No	34.2	164/397 (41.3)	1.0		26/406 (6.4)	1.0		9/395 (2.3)	1.0	
Yes	65.8	248/779 (31.8)	0.7 (0.5–0.9)	0.001	56/781 (7.2)	1.1 (0.7–1.8)	0.6	16/771 (2.1)	0.9 (0.3–1.6)	0.8
*Emotional support*										
*Can talk to family if something troubling you*										
No	6.3	25/74 (33.8)	1.0		8/74 (10.8)	1.0		7/72 (9.7)	1.0	
Yes	93.7	386/1097 (35.2)	1.1 (0.6–1.7)	0.8	72/1108 (6.5)	0.6 (0.3–1.2)	0.2	17/1089 (1.6)	0.1 (0.1–0.4)	< 0.001
*Have someone can talk to if conflict with parents*										
No	46.6	226/546 (41.4)	1.0		45/553 (8.1)	1.0		11/545 (2.0)	1.0	
Yes	53.4	186/630 (29.5)	0.6 (0.5–0.8)	< 0.001	37/634 (5.8)	0.7 (0.4–1.1)	0.12	14/621 (2.3)	1.1 (0.5–2.5)	0.8
*Empowerment*										
*Self-efficacy*										
Low	28.8	141/335 (42.1)	1.0		19/341 (5.6)	1.0		6/335 (1.8)	1.0	
Medium/High	71.2	272/842 (32.3)	0.7 (0.5–0.9)	0.002	63/848 (7.4)	1.4 (0.8–2.3)	0.3	19/834 (2.3)	1.3 (0.5–3.2)	0.6
*Spoken out against gender disadvantage*										
Low	38.6	188/454 (41.4)	1.0		31/458 (6.8)	1.0		10/447 (2.2)	1.0	
Medium/High	61.4	225/723 (31.1)	0.6 (0.5–0.8)	< 0.001	51/731 (7.0)	1.0 (0.7–1.6)	0.9	15/722 (2.1)	0.9 (0.4–2.1)	0.9

^a^Data missing for 56 participants (4.7%). ^2^Data missing for 7 participants (0.6%). ^3^Data missing for 8 participants (0.7%)

#### Psychosocial resources (Table 2)

Information on girl’s access to psychosocial resources was obtained from the face-to-face interviews. Social integration comprised two variables: membership and participation in a group; and made new friends in past year (yes/no). We asked about participation in seven different informal groups (sports, study, life skills, dancing/singing/music, savings, bhajana (singing, dancing), Kishori (adolescent) using a 6 point likert type scale. Response choices were; not a member, member but do not attend, attend less often, attend at least once a month, once a week, or almost every day. Principal Component Analysis (PCA) and Cronbach’s Alpha analyses were used to derive the best set of items from girls’ responses to the membership and participation in informal groups scale which were used in the analyses. Responses for four items (sports, life skills, study, dancing/singing/music group), Cronback’s Alpha (0.63) were used in the analyses. Girls scores for each item were summed (4–24), and the total sample scores divided into tertiles (low, medium, high). For the final analyses, this was coded as a binary variable (low vs. medium / high membership and participation).

Emotional support comprised two variables: can talk to someone in the family if something was troubling you (yes/no); and have someone you could talk to if you experienced conflict with your parents (yes/no). Empowerment comprised two variables: self-efficacy and speaking out against gender disadvantage, both derived using PCA and internal consistency analyses on responses to seven item (If I work hard, I feel capable of achieving my goals; I am optimistic that I will have a better life than my parents; I feel able to talk to my parents about my hopes and aspirations; I can stand up for my right to be treated with the same respect as my brother; I can express my views on marriage even if they differ from those of my parents; I can ask my parents to support my completion of secondary education; I feel able to seek help from others to achieve my goals) and three item (I feel willing and able to speak out… in support of girls education; against child marriage; against eve teasing) scales respectively. Both scales used a three-point likert type response choice (Agree/ somewhat agree/ do not agree). The Chronbach’s Alpha for the responses to the 7 item self-efficacy scale and the 3 item speaking out against gender disadavantage scale were 0.67 and 0.78, respectively. Girls scores for each scale were summed, the total score divided into tertiles, and coded as binary variables (low vs. medium/high) in the analyses.

#### Outcome measures - psychological distress

Psychological distress was measured using three independent outcomes: I have no hope for my future (disagree vs. somewhat agree/agree); Felt down depressed or hopeless (past two weeks) (not at all vs. one or more days); Bothered by thoughts that I would be better off dead, or of hurting myself in some way (past two weeks) (not at all vs. one or more days). The first two outcomes were asked in the face-to-face questionnaire and the last outcome was asked in the self-completed questionnaire. The three items used to measure psychological distress came from different validated scales. “I have no hope for the future” is similar to items used to measure hopelessness in the Beck Hopelessness scale (Item 7. My future seems dark to me) [38]. “Feeling down, depressed or hopeless (past 2 weeks)” and “Bothered by thoughts that I would be better off dead, or of hurting myself in some way (past 2 weeks)” were taken from the 9-item Patient Health Questionnaire (PHQ-9), which is used to assess depression and has been validated for use with adolescents in India [39]. The complete Hopelessness and PHQ-9 scales were not included in the surveys as measuring psychological distress was not a primary aim of the overall study and we did not want to overburden participants with a lengthy questionnaire.

### Analysis

**Fig. 1:**
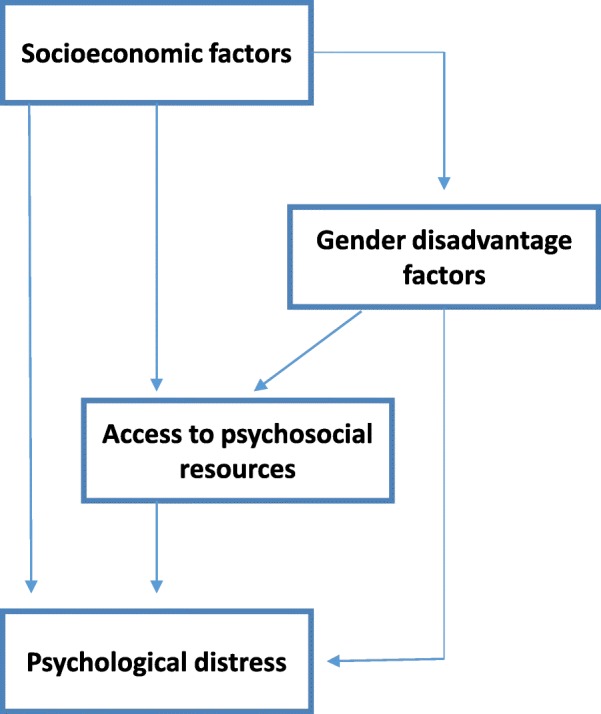
A conceptual framework for the social risk factors for psychological distress among low caste adolescent girls in north Karnataka, south India

Our analyses were guided by a conceptual framework (Fig. 1) postulating a hierarchical relationship between factors associated with psychological distress among adolescent girls in this setting. This model was derived from a review of the literature on gender disadvantage and the aetiology of psychological distress in LMIC, and by our experience of conducting research on the structural drivers of vulnerability and of working with adolescents in this setting. The model formed the basis for assessing the effect of various factors as direct or confounded by other factors. First we examined the unadjusted associations between variables within the three domains (socioeconomic, gender disadvantage, psychosocial resources) and the three outcome measures. Logistic regression was used to estimate odds ratios (OR) and 95% confidence intervals (CI). We built the final multivariate model for each outcome variable, by including all variables associated with *p* ≤ 0.15 with the outcome in the univariate analyses [40]. We first introduced factors from the sociodemographic domain which retained an association with the outcome. We then added factors from the gender disadvantage domain, and finally factors in the psychosocial resources domain, retaining in each step only those factors which remained significant (*p* ≤ 0.05). Multivariate logistic regression was used to estimate adjusted odds ratios (AOR) with 95% CI, and the Wald Test was the statistical test used. We used the likelihood ratio test to examine potential effect modification of psychosocial resources on factors which were significant in the final multivariate models. Analyses were performed using STATA version 14.1.

## Results

Overall, of 1284 SC/ST girls enrolled in class seven (last year of primary school), 1191 girls (response rate 92.6%) participated and completed the interview. Of the 1189 family members who completed the brief household demographic and economic questionnaire, almost two thirds (64.5%) were illiterate. The majority were female (63.3%) and most were parents (85.8) or grandparents (7.7%) (with the remainder (6.6%) siblings, cousins, uncles, aunts etc).

### Sample characteristics

The median age of girls was 13 years (IQR 13–13; range 11–18 years). Most were from scheduled castes (77.3%) and 16% were orphaned by one or both parents. Participants had a median of 3 siblings (IQR 2–4; range 0–9) and many (63.3%) were from households with an illiterate household head. 16.7% had parents who had migrated for work in the past year and a small proportion of girls (8.4%) had migrated for work in the past year, with or without their family. Participants were more likely to be from Bagalkote (57.2%) than Vijayapura district.

### Prevalence of psychological distress

Response rates for psychological distress were 98.8% (no hope for the future), 99.8% (down, depressed or hopeless, past 2 weeks) and 98.2% (suicidal ideation, past 2 weeks). Overall, 35.1% of girls reported no hope for the future, 6.9% said they had felt down, depressed or hopeless in the past 2 weeks, and 2.1% reported suicidal ideation in the past 2 weeks (Table 1).

### Association with socioeconomic factors

Univariate analysis of socioeconomic factors (Table 1) found having no hope for the future was associated with orphan-hood, having an illiterate household head, living in a household with a medium wealth index, and living in Bagalkote district. Feeling down, depressed or hopeless in the past two weeks was associated with living in Vijayapura district, and there was a trend for being from a scheduled caste compared with a scheduled tribe. Girls who reported suicidal ideation in the past 2 weeks were likely to be older (14+ years), and there was a trend for increasing prevalence with an increasing number of siblings.

### Association with gender disadvantage factors

Reported exposure to violence and harassment was low; 1.6% of girls reported previous sexual abuse (4.7% did not answer this question), 8.0% reported recent eve-teasing experience, and 4.1% reported a harassing or bullying environment at their current or last school (Table 2). Girls who did not answer the sexual abuse question had similar sociodemographic characteristics to the non-abused girls and were also more likely to have not answered the question on recent eve-teasing (data not shown). Nearly two thirds (61.5%) of participants had started menstruating. Most girls (95.3%) were unmarried, with the remainder engaged (1.1%) or married (3.6%). While most girls (84.6%) were regularly attending secondary school, 7.4% had been frequently absent in the previous month and 8.0% had dropped out of school (Table 2).

Univariate analysis of gender disadvantage factors found that having no hope for the future was associated with not having started menstruating and recent eve-teasing, and there were trends for being engaged, and having dropped out of secondary school. Feeling down, depressed or hopeless in the past two weeks was also associated with recent eve-teasing, frequent absenteeism and having dropped out of secondary school; it was also associated with a harassing or bullying school environment. Girls who reported suicidal ideation in the past 2 weeks were more likely to report previous sexual abuse and to have dropped out of secondary school.

### Association with psychosocial resources

When we examined associations between psychological distress and access to psychosocial resources (Table 2), having no hope for the future was associated with poor social integration (membership and group participation; making new friends in the past year), poor emotional support (not having someone to talk to if experienced conflict with parents), and low empowerment (low self-efficacy; not speaking out against gender disadvantage). There were no associations between feeling down, depressed or hopeless in the past two weeks and access to any of the psychosocial resources measured. Girls who reported suicidal ideation in the past 2 weeks were significantly more likely to report poor parental emotional support (not having anyone in the family to talk to if something was found troubling her), compared with girls who did not report suicidal ideation.

### Final multivariate model

In the final multivariate models (Table 3) having no hope for the future was independently associated with socioeconomic factors (orphan-hood, being from a wealthier family, living in Bagalkote district), gender disadvantage (not starting menarche, recent eve-teasing (among girls who had reached menarche)), and lack of access to psychosocial resources (not being a member or participating in informal groups, not making any new friends in the past year and having nobody to talk to if experienced conflict with parents). There was no evidence for a buffering effect of access to psychosocial resources on having no hope for the future.

**Table 3:** Final multivariate model of risk factors for psychological distress among adolescent girls (aged 13–14 years) in north Karnataka, south India, 2014

Risk Factor	Do not have hope for the future		Feeling down, depressed or hopeless (past 2 weeks)		Suicidal thoughts (past 2 weeks)	
	Adjusted OR (95% CI)	*P* value	Adjusted OR (95% CI)	*P* value	Adjusted OR (95% CI)	*P* value
Socioeconomic factors						
*Orphan hood* (Ref – no)	–					
Yes	1.4 (1.0–2.0)	0.05				
*Literate household head* (Ref – no)	–					
Literate	0.8 (0.6–1.0)	0.08				
*Household wealth index* (Ref – poorest)	–					
Medium	1.7 (1.2–2.3)	0.002				
Richest	1.5 (1.1–2.0)	0.02				
District (Ref – Bagalkote)	–		–			
Vijayapura	0.6 (0.4–0.7)	< 0.001	2.5 (1.6–4.1)	< 0.001		
Gender Disadvantage						
*Started Menstruating* (Ref – no)	–					
Yes	0.7 (0.5–0.9)	0.003				
*Sexual abuse ever* (Ref – no)					–	
Yes					11.9 (3.0–47.0)	< 0.001
*Recent eve teasing* (Ref – no)	–		–			
Yes	1.5 (1.0–2.4)	0.07	2.9 (1.6–5.4)	0.001		
Pre-Menarche (Ref – no)	0.7 (0.3–1.9)	0.5	0.9 (0.1–7.4)	0.9		
Menarche (Ref – no)	1.9 (1.2–3.3)	0.01	3.5 (1.8–7.0)	< 0.001		
*Harassing/abusive school environment* (Ref – no)						
Yes			3.9 (1.8–8.2)	< 0.001		
*Marital status* (Ref – Unmarried)	–					
Engaged	2.9 (0.9–9.7)	0.08				
Married	0.9 (0.4–1.7)	0.7				
*Schooling status* (Ref – In school)			–			
Frequent absenteeism			2.8 (1.5–5.5)	0.002		
Dropped out of school			2.1 (1.0–4.3)	0.05		
Psychosocial resources						
*Social Integration*						
*Membership and participation in informal groups* (Ref –None/ Low)	–					
Medium/high	0.5 (0.4–0.6)	< 0.001				
*New friends past year* (Ref – no)	–					
Yes	0.7 (0.6–1.0)	0.03				
*Emotional support*						
*Can talk to family if something troubling you* (Ref – no)					–	
Yes					0.2 (0.1–0.5)	0.002
*Have someone can talk to if conflict with parents* (Ref – no)	–					
Yes	0.6 (0.4–0.7)	< 0.001				

Final models were built for each outcome variable by including all variables associated p ≤ 0.15 with the outcome in the univariate analyses. We first introduced factors from the socioeconomic domain, and then added factors from the gender disadvantage domain, and finally the psychosocial buffer domain, retaining in each step only those factors which remained significant (p ≤ 0.05)

Feeling down, depressed or hopeless in the past two weeks was independently associated with gender disadvantage factors (recent eve-teasing (among girls who had reached menarche), a harassing or bullying current or past school environment, and frequent school absenteeism / school drop-out), and living in Vijayapura district. There was no evidence for a buffering effect of access to psychosocial resources on this outcome.

Suicidal ideation in the past two weeks was independently associated with experiencing sexual abuse and lack of parental emotional support (Table 3). There was insufficient power to examine the potential buffering effects of access to psychosocial resources on suicidal ideation.

## Discussion

In this study involving 13–14 year-old low caste adolescent girls, we found high levels of psychological distress and strong associations with gender disadvantage factors (school drop-out, child marriage, sexual abuse, and harassment) common to girls in many LMIC. In addition, having no hope for the future and recent suicidal ideation were also associated with a lack of access to psychosocial resources. Taken together, the results are among the first to examine the correlates of psychological distress among early adolescent girls in an LMIC setting.

Suicidal ideation refers to thoughts of harming or killing oneself and can be a precursor for attempted and completed suicide [9, 10]. Understanding the risk factors is important for prevention. The prevalence of suicidal ideation in our study (2.1%) was comparable with a study in Goa (3.9%) among older youth (16–24 years) [8] and broadly similar to the pooled prevalence (10.7%) for suicide ideation among > 58,000 youth (13–17 years) from seven south-east Asia and Western Pacific countries, which used a time frame of past year (vs. past 2 weeks) [11]. Although suicide in India gains significant media coverage, it is highly stigmatised and illegal, and is likely to have been under-reported in our study. Nonetheless, we found that it was independently associated with sexual abuse and a lack of emotional support. Associations between physical and sexual abuse and suicidal behaviour among adolescents are well established in the literature, especially from high-income countries [8, 41]. In addition, loneliness and lack of parental support were two key predictors of suicide ideation among youth in south-east Asia and Western Pacific countries [11].

The suicidal ideation question included in the PHQ-9 measures ‘passive’ (Bothered by thoughts that you would be better off dead, or of hurting yourself in some way (past 2 weeks)) as opposed to ‘active’ (e.g. Have you actually had any thoughts of killing yourself; Have you been thinking about how you might do this? [42]) suicidal ideation. The indicator used in the PHQ-9 is used in several other screening tools (see [43] for examples) and recent research using longitudinal data from adults in the general population in a high-income setting, suggests this item is a strong predictor of subsequent suicide attempt and suicide death [10]. The PHQ-9 was designed to assess depression severity, not to assess suicide risk. While frequent thoughts of death or self-harm are strongly associated with subsequent suicidal behaviour, there is a trade-off between sensitivity (identifying all individuals at risk) and positive predictive value (identifying those at highest risk) [10]. Sensitivity and positive predictive value are highest when the time frame is short (e.g. past month vs. past 2 years) [10]. Broader questions regarding hopelessness or suicidal thoughts, such as those in the Beck Depression Inventory or the Quick Inventory of Depressive Symptomatology might prove more sensitive [44, 45]. Questions focused on suicidal ideation such as the Colombia Severity Rating Scale or the Beck Scale for Suicidal Ideation might identify a smaller group at higher risk [42]. Despite these limitations, we focused on the PHQ-9 indicator, not because it is the optimal measure, but because it is widely used in community practice and because the short length made it more practical for our survey tool.

Experiencing a harassing or bullying schooling environment (current or most recent school), being frequently absent in the past 30 days, or having dropped out of school, were key factors independently associated with feeling down, depressed or hopeless in the past 2 weeks. With cross-sectional data such as these, it is not possible to ascertain the direction of causality but our qualitative research and research from elsewhere suggests that education is usually highly valued among girls, who understand the benefits for themselves and the next generation [23, 30]. Unless girls are exceptionally committed to their education and are supported by key figures within their family, school or community, they usually have little control over the ‘upstream’ determinants of dropping-out, such as their increasing household responsibilities, the economic need to be contributing financially, the ‘visible’ preservation of their sexual purity (i.e. through restricted movement), and their marriage [23]. Interestingly, recent eve-teasing was also independently associated with recent depressive thoughts. Our qualitative research suggests that the ‘fall-out’ of eve-teasing in terms of restrictions on a girl’s movements, ending her education, questioning her sexual purity (and therefore ‘honour’), and bringing forward her marriage, are far more pervasive than any actual physical or sexual harm experienced from eve-teasing itself [32]. Addressing these key gender disadvantage factors should improve the health and well-being of girls in this setting.

One-third of the study population reported that they did not have hope for the future. This was independently associated with recent eve-teasing (among those who had reached menarche), and there was a trend towards an association with being engaged. Given the social repercussions of being eve-teased, especially among girls who have reached menarche, and given the dramatic changes in a girl’s life that accompany marriage (moving to the spouse’s home and village; withdrawal from education; child-bearing, etc.), these associations are not surprising. Instead it underscores the importance of challenging harmful gender norms around sexual purity, family honour and child marriage, as well as creating an environment that enables dialogue and communication between male and female children and adolescents, so as to remove eve-teasing as the only mechanism for adolescent boys and girls to communicate. Having no hope for the future was also independently associated with socioeconomic factors (orphan-hood, living in Bagalkote district and being from a wealthier family), and with a lack of access to psychosocial resources (not being a member or participating in informal groups, not making any new friends in the past year, having nobody to talk to if conflict was experienced with parents). Social integration and its links with mental health is well established [46]; indeed social inclusion - along with freedom from discrimination and violence, and access to economic resources – has been identified as a key social and economic determinant of community and individual mental health [47].

The current study has several limitations. With cross-sectional survey data such as these, it is not possible to ascertain the direction of associations or to make causal inferences from the results. We were not able to measure depression at baseline, and so were not able to determine when feelings of sadness and losing interest became a disorder by distinguishing the number, duration and impact of these experiences in a given time frame [5]. We have amended our end-line survey tool to include validated tools to measure depression and anxiety. In addition, sexual harassment and eve teasing questions could have been improved if we had described what specific behaviours we meant by these terms. Although girls provided informed assent to participate in the study and interviews were conducted in private at their home or place of their choice, participants in our study were young (13–14 years) and fears around confidentiality, together with social desirability bias, are likely to have resulted in under-reporting of sensitive topics such as marital status, sexual abuse, eve-teasing experience, suicidal ideation and potentially the two other mental health outcomes as well. Together, this means that the values presented here are likely underestimates of the true prevalence and levels of association between gender disadvantage and psychosocial distress. Responses to the brief household questionnaire (caste, orphan hood, household composition, literacy status, and household assets) may have differed according to the respondent’s gender and age (grandparent vs. parent); for example, reporting on household assets may have been influenced by social desirability bias among men. Low literacy levels among some participants may have resulted in measurement error (misclassification bias) with respect to variables assessed in the self-completed questionnaire. We attempted to mitigate this by training interviewers to work through the self-completed questionnaire item by item with participants and by simplifying potential answers to binary (yes/no). The small numbers for some variables such as suicidal ideation, resulted in wide confidence intervals, and may have missed factors weakly associated with this outcome.

## Conclusions

The results of this study are among the first to examine the correlates of psychological distress among early adolescent girls in an LMIC setting. A third of low caste girls in north Karnataka have no hope for the future, with some contemplating suicide. Interventions are needed which address the upstream structural and gender norms-based determinants of poor mental health (poverty, gender norms around sexual purity and reputational risk, girls lack of autonomy, low status, vulnerability to sexual abuse and harassment) and provide adolescent friendly and accessible services for girls who require treatment and support.

## Additional file

Additional file 1: Samata baseline survey tool for girls Cohort 2. The questionnaire for girls comprises three parts: a brief demographic and economic questionnaire asked to an adult family member; a structured questionnaire asked by a female interviewer to adolescent girls; and an anonymous short self-completed questionnaire containing sensitive questions that girls completed by themselves (or in the case of low literacy levels, with the assistance of interviewers). (PDF 433 kb)

## Abbreviations

AOR: Adjusted Odds Ratio
CI: Confidence Intervals
IQR: Inter-Quartile Range
LMIC: Low and Middle Income Countries
OR: Odds Ratio
PCA: Principal Component Analysis
RCT: Randomised Control Trial
SC/ST: Scheduled Caste / Scheduled Tribe
