# Supplementary material for: Prevalence and correlates of psychological distress among 13–14 year old adolescent girls in North Karnataka, South India: a cross-sectional study
Source: BMC Public Health. 2019 Jan 10;19:48. doi: 10.1186/s12889-018-6355-z (PMC6327490; doi:10.1186/s12889-018-6355-z)
Supplement: Supplementary file 1 — Samata baseline survey tool for girls Cohort 2. The questionnaire for girls comprises three parts: a brief demographic and economic questionnaire asked to an adult family member; a structured questionnaire asked by a female interviewer to adolescent girls; and an anonymous short self-completed questionnaire containing sensitive questions that girls completed by themselves (or in the case of low literacy levels, with the assistance of interviewers). (PDF 433 kb) [file 12889_2018_6355_MOESM1_ESM.pdf]

CONFIDENTIAL: *FOR RESEARCH PURPOSE ONLY*

STRIVE
Tackling the structural drivers of HIV

EVALUATION OF AN INTERVENTION SUPPORTING ADOLESCENT GIRLS FROM MARGINALIZED COMMUNITIES TO STAY IN SCHOOL, REDUCE EARLY MARRIAGE, AND DELAY ENTRY INTO SEX WORK

Study with SC/ST Adolescent Girls

KARNATAKA HEALTH PROMOTION TRUST, BANGALORE

SECTION I: INTERVIEW INFORMATION

Study Number: ________________________ [ ][ ][ ][ ]

Name of the district: ________________________ [ ][ ]

Name of Taluk: ________________________ [ ][ ]

Name of City/Town/ Village ________________________ [ ][ ][ ]

Date of interview:

DAY [ ][ ] MONTH [ ][ ] YEAR [ ][ ][ ][ ]

Name and code of interviewer: ________________________ [ ][ ]

Consent Status: ________________________ [ ]

Parents/guardians given consent for the interview	= 1
Did not give consent for interview	= 2
Not available	= 3

Completion Status: ________________________ [ ]

Completed interview	= 1
Partially completed interview	= 2
Not eligible	= 3
Refused	= 4
Not available	= 5

SECTION II: EDITING AND DATA ENTRY

Name and code of Supervisor: ________________________ [ ]

Date of scrutinizing the questionnaire:

DAY [ ][ ] MONTH [ ][ ] YEAR [ ][ ][ ][ ]

Signature of Supervisor: ________________________

Name and code of Data entry person: ________________________ [ ]

Date of data entry: DAY [ ][ ] MONTH [ ][ ] YEAR [ ][ ][ ][ ]

Signature of data entry person: ________________________

SECTION III: DEMOGRAPHIC CHARACTERISTICS

ASK THIS SECTION (I.E. UPTO QN.327) TO THE ADULT FAMILY MEMBER OF THE ADOLESCENT GIRL SELECTED FOR INTERVIEW FROM THE AY 2013-14 CLASS 7 ROSTER

I'd like to begin by asking you about the people who usually live in your household, including those living here currently and those who may temporarily be away. I am especially interested in the parents and siblings of [name] the adolescent girl selected for interview, so I would like you to include them if they are alive, even if they do not live in your household.

LINE NO.	USUAL RESIDENTS	RELATIONSHIP TO THE AG SELECTED FOR INTERVIEW	SEX	RESIDENCE		AGE	MARITAL STATUS	EDUCATION			OCCUPATION	DEVADASI STATUS	SEX WORKER STATUS
							(IF AGE 10 OR OLDER)10	IF AGE 5 YEARS OR OLDER5		IF AGE<25		FOR FEMALE ONLY	
	Please give me the names of the persons who usually live in your household starting with the name of the respondents name	What is the relationship of (name) to the AG?	Is (name) male or female	Does (name) usually live here?	Did (name) stay here last night?	How old is (name)?	What is the current marital status of (name)?	Can (name) read and write?	What is the highest standard (name) has completed?	Is (Name) currently in School/ College?	What is the occupation of (Name)?	Is (Name) a Devadasi?	Is (Name) a Sex worker?
(1)	(2)	(3)	(4)	(5)	(6)	(7)	(8)	(9)	(10)	(11)	(12)	(13)	(14)
		USE CODE	M F	YES NO	YES NO	IN YEARS	USE CODE	YES NO	STANDARD	YES NO	USE CODE	YES NO	YES NO
1			1 2	1 0	1 0			1 0		1 0		1 0	1 0
2			1 2	1 0	1 0			1 0		1 0		1 0	1 0
3			1 2	1 0	1 0			1 0		1 0		1 0	1 0
4			1 2	1 0	1 0			1 0		1 0		1 0	1 0
5			1 2	1 0	1 0			1 0		1 0		1 0	1 0
6			1 2	1 0	1 0			1 0		1 0		1 0	1 0
7			1 2	1 0	1 0			1 0		1 0		1 0	1 0
8			1 2	1 0	1 0			1 0		1 0		1 0	1 0
9			1 2	1 0	1 0			1 0		1 0		1 0	1 0
10			1 2	1 0	1 0			1 0		1 0		1 0	1 0

(1)	(2)	(3)	(4)	(5)	(6)	(7)	(8)	(9)	(10)	(11)	(12)	(13)	(14)
11			1 2	1 0	1 0			1 0		1 0		1 0	1 0
12			1 2	1 0	1 0			1 0		1 0		1 0	1 0
13			1 2	1 0	1 0			1 0		1 0		1 0	1 0
14			1 2	1 0	1 0			1 0		1 0		1 0	1 0

A. CODES FOR COLUMN (3), RELATIONSHIP TO THE AG: 01= ADOLESCENT GIRL 02=PARENT, 03=PARENT-IN-LAW, 04=BROTHER/SISTER, 05=BROTHER-IN-LAW/SISTER-IN-LAW 06=SPOUSE, 07=NIECE/NEPHEW, 08=SON/DAUGHTER, 09=GRAND PARENT, 10=OTHER RELATIVE, 11=NOT RELATED

B.CODES FOR COLUMN (7) AGE: AGE LESS THAN 1 YEAR=00, AGE 95 YEARS OR MORE=95, DON'T KNOW=98

C.CODES FOR COLUMN (8), CURRENT MARITAL STATUS: 01=CURRENTLY MARRIED, 02=MARRIED, BUT GAUNA NOT PEROFRMED,03=WIDOWED, 04=DIVORCED, 05=SEPERATED, 06=DESERTED, 07=NEVER MARRIED, 98=DON'T KNOW

D.CODE FOR COLUMN (10), HIGHEST STADARD COMPLETED: IF ILLITERATE, RECORD 0, OTHERWISE, RECORD HIGHEST GRADE COMPLETED

10E.CODE FOR COLUMN (12) OCCUPATION: 01=CULTIVATOR, 02=AGRICULTURAL LABOURER, 03=NON-AGRICULTURAL LABOURER, 04=BUSINESS, 05=SALARIED EMPLOYMENT, 06=HOUSEWORK, 07=STUDENT, 08=NOT WORKING/UNEMPLOYED, 09=SEX WORK, 10=OTHERS.

NO.	QUESTIONS	CODING CATEGORIES	SKIP
315	What is the name of the head of your household?<br>(CHECK THE NAME TOLD IN THE HOUSEHOLD LIST ABOVE AND REOCRD THE LINE NUMBER OF THE PERSON)	LINE NUMBER OF HOUSEHOLD HEAD .... [ ][ ]	
316	Does a member of this household own this house?	YES .... 1<br>NO .... 0<br>DON'T KNOW 98	
317	**OBSERVATION: INTERVIEWER: DO NOT ASK**<br>IS THE HOME KUCCHA, SEMI-PUCCA OR PUCCA?	KUCCHA .... 1<br>SEMI-PUCCA .... 2<br>PUCCA 3	
318	Does this household own any agricultural land?	YES .... 1<br>NO .... 0<br>DON'T KNOW 98	NO, DON'T KNOW → 320
319	How much agricultural land does this household own? ______________<br>{size and unit}	ACRES .... [ ][ ][ ] .. [ ][ ]<br>DON'T KNOW 9998	
320	How many rooms are there in your house including kitchen, but excluding toilets and bathrooms if any?	NUMBER OF ROOMS .... [ ][ ]	
321	What kind of toilet facility does your household have?	NO FACILITY .... 0<br>OWN FLUSH TIOLET .... 1<br>SHARED FLUSH TOILET .... 2<br>OWN PIT TOILET .... 3<br>SHARED PIT TOILET .... 4<br>OTHER (SPECIFY) 96	
322	What is the **main** source of lighting for your household?	ELECTRICITY .... 1<br>KEROSENE .... 2<br>GAS .... 3<br>OIL .... 4<br>OTHERS(SPECIFY) 96	
323	What is the main type of fuel your household commonly uses for cooking?	WOOD/CROP RESIDUE/ DUNG CAKES/ COAL CHARCOAL ...1<br>KEROSENE ....2<br>ELECTRICITY ....3<br>LIQUID PETROLEUM GAS (LPG) ....4<br>BIO-GAS ....5<br>OTHERS(SPECIFY) 96	
324	What is the **main** source of drinking water for the members of your household?	**OWN**<br>PIPED WATER/HANDPUMP/COVERED WELL ....1<br>OPEN WELL ....2<br>**PUBLIC**	

NO.	QUESTIONS	CODING CATEGORIES	SKIP
		PIPED WATER/HANDPUMP/COVERED WELL ....3<br>OPEN WELL ....4<br>SURFACE WATER (SPRING/RIVER/STREAM/POND/ LAKE/DAM ....5<br>RAINWATER ....6<br>TANKER TRUCK ....7<br>OTHER (SPECIFY) ___________________ 96	
325	Does your household own any of the following assets?<br><br>**(READ EACH ITEM AND RECORD RESPONSES FOR EACH)**	**YES** **NO**<br>A. ELECTRICITY .... 1 0<br>B. ELECTRIC FAN .... 1 0<br>C. BICYCLE .... 1 0<br>D. MOTORCYCLE/SCOOTER ....1 0<br>E. COT/BED .... 1 0<br>F. RADIO/TRANSISTOR .... 1 0<br>G. B&W TELEVISION .... 1 0<br>H. COLOUR TELEVISION .... 1 0<br>I. LAND LINE/MOBILE .... 1 0<br>J. SEWING MACHINE .... 1 0<br>K. COMPUTER/LAPTOP .... 1 0<br>L. REFRIGERATOR .... 1 0<br>M. WATCH/CLOCK .... 1 0<br>N. ANIMAL DRAWN CART .... 1 0<br>O. CAR/TRUCK .... 1 0<br>P. WATER PUMP .... 1 0<br>Q. TRESHER .... 1 0<br>R. TRACTOR 1 0	
326	What is average monthly income of your household from all the sources?	RUPEES .... [ ][ ][ ][ ][ ]<br>DON'T KNOW ....98<br>NO ANSWER/.....................................99	
327	In the past 4 weeks, how often did you or any member of your household go to sleep at night hungry because there was not enough food?	NEVER ....1<br>RARELY OR SOMETIMES(1-5 TIMES) ....2<br>OFTEN (MORE THAN 5 TIMES) 3	
328	What is your caste or tribe?	SCHEDULED CASTE......... .... 1<br>SCEDULED TRIBE .... 2<br>OTHER BACKWORD CLASS .... 3<br>ANY OTHER (SPECIFY) .... 96<br>DON'T KNOW 98	3, 96, 98 → **END INTER-VIEW**
	**FAMILY MIGRATION**		
328a	Did the family (parents) of [AG name] ever migrate outside the district for work in the past 12 months?	YES ....1<br>NO ....0<br>DON'T KNOW ....98<br>NO ANSWER 99	0, 98, 99 → 329

NO.	QUESTIONS	CODING CATEGORIES	SKIP
328b	Which district and state did the family of [AG name] migrate in the past 12 months?	A. DISTRICT__________________________________<br>B. STATE____________________________________	

ASK THE FOLLOWING QUESTIONS (QN. 329 ONWARDS) TO THE ADOLESCENT GIRL SELECTED FOR INTERVIEW IN COMPLETE PRIVACY

NO.	QUESTIONS	CODING CATEGORIES	SKIP
	**I'd like to begin our interview by asking some basic questions about your current life situation.**		
329	Where do you currently live?	AT HOME WITH ONE OR MORE OF YOUR BIRTH PARENTS ....1<br>WITH THE FAMILY OF YOUR HUSBAND OR HUSBAND TO BE ....2<br>IN THE HOME OF ANOTHER FAMILY MEMBER ....3<br>ELSEWHERE [NOTE WHERE] 4	
330	What is your current marital status?	CURRENTLY MARRIED .... 1<br>MARRIED BUT GAUNA NOT PERFORMED .... 2<br>DESERTED/SEPARATED/DIVORCED .... 3<br>WIDOWED .... 4<br>ENGAGED .... 5<br>NEVER MARRIED .... 6<br>DON'T KNOW .... 98<br>NO ANSWER 99	
331	Are you a Devadasi?	YES .... 1<br>NO .... 0<br>DON'T KNOW .... 98<br>NO ANSWER 99	
332	CHECK Q 330:CODE '1','2','3','4' CIRCLED ☐ ↓	CODE '5','6','98','99' CIRCLED ☐ →	335
333	How old were you at the time of your marriage?	AGE IN COMPLETED YEARS .... ☐☐<br>DON'T KNOW 98	
334	How old were you when you started living with your husband?	AGE IN COMPLETED YEARS .... ☐☐<br>HAVEN'T STARTED LIVING WITH HUSBAND .... 95<br>DON'T KNOW 98	

Q335	Now I would like to ask some details of your siblings. Can you please give me the details of your siblings starting with the eldest sibling?										
**LINE NO.**	**Siblings**	**SEX**	**AGE**	**EDUCATION**			**MARITAL STATUS**	**RESIDENCE**			**Remark (Record survival status)**
				**IF AGE 5 YEARS OR OLDER5**		**IF AGE<25**	**(IF AGE 10 OR OLDER)10**				
	**Please give me the names of your siblings starting with eldest sibling.**	**Is (name) male or female**	**How old is (name)?**	**Can (name) read and write?**	**What is the highest standard (name) has completed?**	**Is (Name) currently in School/ College?**	**What is the current marital status of (name)?**	**Does (name) currently live with your family?**	**If 'no' which state they are living in now?**	**If 'no' which district they are living in now?**	
**(1)**	**(2)**	**(3)**	**(4)**	**(5)**	**(6)**	**(7)**	**(8)**	**(9)**	**(10)**	**(11)**	**(12)**
	Name	M F	IN YEARS	YES NO	IN YEARS	YES NO	USE CODE	YES NO	STATE NAME	DISTRICT NAME	
1		1 2		1 0		1 0		1 0			
2		1 2		1 0		1 0		1 0			
3		1 2		1 0		1 0		1 0			
4		1 2		1 0		1 0		1 0			
5		1 2		1 0		1 0		1 0			
6		1 2		1 0		1 0		1 0			

A.CODES FOR COLUMN (4) AGE: AGE LESS THAN 1 YEAR=00, DON'T KNOW=98
B.CODES FOR COLUMN (6) HIGHEST STADARD COMPLETED: IF ILLITERATE, RECORD 0, OTHERWISE, RECORD HIGHEST GRADE COMPLETED
C.CODES FOR COLUMN (8), CURRENT MARITAL STATUS: 01=CURRENTLY MARRIED, 02=MARRIED, BUT GAUNA NOT PEROFRMED,03=WIDOWED, 04=DIVORCED, 05=SEPERATED, 06=DESERTED, 07=NEVER MARRIED, 98=DON'T KNOW

	**BLOCK IV: ENTRY INTO SECONDARY SCHOOL AND QUALITY EDUCATION**<br>Now I'd like to ask you a few questions about your school and your schooling experience.		
**NO.**	**QUESTIONS**	**CODING CATEGORIES**	**SKIP**
401	What was the last standard you completed?	STANDARD .... ☐☐	
402	Was the last school located in the same village where you live?<br>If "No" can you please tell me the name of village and taluk where you attended last standard?	YES .... 1<br>NO .... 0<br>**IF NO, THEN**<br>VILLAGE NAME______________________________<br>TALUK NAME___________________________________	
403	Are you still attending school?	YES .... 1<br>NO 0	0 → **405**
404	In which standard are you currently studying?	STANDARD .... ☐☐	
	**SCHOOL ABSENTEEISM**		
404a	Thinking about the last month you went to school, how many days in that month would you say that you didn't attend classes?	NUMBER OF DAYS DID NOT ATTEND CLASS . ☐☐<br>DIDN'T MISS ANY CLASS IN THE LAST MONTH . 95	95 → **407**
404b	Thinking about the last week you went to school, how many days would you say that you didn't attend?	NUMBER OF DAYS DID NOT ATTEND CLASS . ☐☐<br>DIDN'T MISS ANY CLASS IN THE LAST WEEK .. 95	
404c	What are the different reasons for not attending the school on some days in the last month?<br><br>(DO NOT READ THE RESPONSE, PROBE FOR MORE)	TO LOOK AFTER YOUNGER SIBLINGS .... A<br>TO DO HOUSEHOLD CHORES<br>.. B<br>TO WORK ON FARM/FIELDS .... C<br>MIGRATION FOR WORK WITHIN DISTRICT ...... D<br>MIGRATION FOR WORK OUTSIDE DISTRICT WITHIN STATE .... E<br>MIGRATION FOR WORK OUTSIDE STATE .... F<br>SICKNESS .... G<br>OTHER (SPECIFY)<br>_____________________________ .... X<br>DIDN'T MISS ANY CLASS IN THE LAST MONTH . 95	A–X → **407**
405	When did you drop-out of school?<br><br>By drop-out we mean when was the first time you did not attend school for 3 or more months	DURATION SINCE DROP-OUT IN MONTHS ☐☐<br>DON'T REMEMBER 98	
406	What is the main reason for drop-out of school?<br><br>**(Do not read the options)**	SCHOOL TO FAR AWAY .... 1<br>TRANSPORT NOT AVAILABLE .... 2<br>I DO NOT WANT TO .... 3<br>PARENTS/GUARDIAN DO NOT WANT ME TO ...... 4<br>HUSBAND DOES NOT WANT ME TO .... 5<br>REQUIRED FOR HOUSEHOLD WORK 6<br>REQUIRED FOR WORK ON FARM/FAMILY BUSINESS 7<br>REQUIRED FOR OUTSIDE WORK FOR PAYMENT IN CASH OR KIND .... 8<br>COSTS TOO MUCH .... 9<br>NO PROPER SCHOOL FACILITIES FOR GIRLS .. 10<br>NOT SAFE TO SEND GIRLS 11	

		NO FEMALE TEACHER .... 12<br>REQUIRED FOR CARE OF SIBLINGS .. 13<br>GOT MARRIED .... 14<br>DID NOT GET ADMISSION/ .... 15<br>HAD PROBLEMS WITH A TEACHER 16<br>HARASSED OR HAD PROBLEMS WITH OTHER STUDENTS .... 17<br>OTHER (SPECIFY) .... 96<br>DON'T KNOW 98	
407	In your opinion, how likely is it that you will complete the entire school year next year?	VERY LIKELY .... 1<br>SOMEWHAT LIKELY .... 2<br>NOT AT ALL LIKEY .... 3<br>DON'T KNOW 98	
408	In your opinion, how likely is it that you will complete 10th standard?	VERY LIKELY .... 1<br>SOMEWHAT LIKELY .... 2<br>NOT AT ALL LIKEY .... 3<br>DON'T KNOW 98	
	**MIGRATION AND WORK**		
408a	Do you do work in the home, such as look after siblings, household chores, etc?	YES .... 1<br>NO 0	→ **408d**
408b	If yes, on the last school day you went to school, how many hours of housework, washing, tending animals, or caring for relatives did you do?	NUMBER OF HOURS .... [ ][ ]<br>DON'T KNOW 98	
408c	On the last school day you did not go to school, how many hours of housework, washing, tending animals, or caring for relatives did you do?	NUMBER OF HOURS .... [ ][ ]<br>DIDN'T MISS ANY CLASS ..... 95<br>DON'T KNOW 98	
408d	Have you ever engaged in a work where you were paid either in the form of money or in kind?	YES .... 1<br>NO .... 0<br>NO ANSWER 99	NO/NO ANSWER → **408g**
408e	Have you ever engaged in a work where you were paid either in the form money or in kind in the past 12 months?	YES .... 1<br>NO .... 0<br>NO ANSWER 99	NO/NO ANSWER → **408g**
408f	What kinds of work were you engaged in the past 12 months?	GRAPE CUTTING .... A<br>SUGAR CANES CUTTING .... B<br>HARVESTING .... C<br>WEEDING .... D<br>SOWING .... E<br>PICKING COTTON .... F<br>CONSTRUCTION WORK .... G<br>OTHERS (SPECIFY) X	
408g	Have you ever travelled outside the district alone or with your family for work?	YES .... 1<br>NO .... 0<br>NO ANSWER 99	NO/NO ANSWER → **408j**

NO.	QUESTIONS	CODING CATEGORIES	SKIP
408h	In the past 12 months, have you ever travelled outside the district alone or with your family for work?	YES .... 1<br>NO .... 0<br>NO ANSWER 99	(NO, NO ANSWER) 408j
408i	In the last 12 months, when you travelled outside the district because either you or your family were working, have you had an occasion where you missed the school?	YES .... 1<br>NO .... 0<br>DROPPED SCHOOL BEFORE LAST 12 MONTHS .... 95<br>NO ANSWER 99	
**NO.**	**QUESTIONS**	**CODING CATEGORIES**	**SKIP**
408j	Have you ever travelled within the district alone or with your family for work?	YES .... 1<br>NO .... 0<br>NO ANSWER 99	(NO, NO ANSWER) 408m
408k	In the past 12 months, have you ever travelled within the district alone or with your family for work?	YES .... 1<br>NO .... 0<br>NO ANSWER 99	(NO, NO ANSWER) 408m
408l	In the last 12 months, when you travelled within the district because either you or your family were working, have you had an occasion where you missed the school?	YES .... 1<br>NO .... 0<br>DROPPED SCHOOL BEFORE LAST 12 MONTHS .... 95<br>NO ANSWER 99	
408m	Approximately how many days in the last 12 months have you missed school because you or your family were working for money?	NUMBER OF DAYS MISSED CLASS .... [ ][ ]<br>DID NOT MISS SCHOOL .... 94<br>DROPPED SCHOOL BEFORE LAST 12 MONTHS .... 95<br>DON'T KNOW 98	
408n	On the last school day you missed because you were working for money, how many hours of work did you do?	NUMBER OF HOURS .... [ ][ ]<br>DID NOT MISS SCHOOL .... 95<br>DON'T KNOW 98	
	**KNOWLEDGE OF GIRLS WORKING IN MAJOR CITIES ELSEWHERE**		
408o	Sometimes girls from villages in this area go to the Sangli, Satara, Solapur, Mumbai, Thane and Pune districts.<br>Do you know of any girls in your village who have gone to these districts to work in the past 12 months?	YES .... 1<br>NO .... 0<br>DON'T KNOW 98	(NO, DON'T KNOW) 409
408p	If yes, which cities have they gone to in the past 12 months?	CITY NAME 1 . ________________________<br>CITY NAME 2 . ________________________<br>CITY NAME 3 . ________________________	
408q	If yes, do you know what kind of work they do?	AGRICULTURAL LABOUR .... A<br>NON AGRICULTURAL LABOUR .... B<br>GARMENTS WORK .... C<br>CONSTRUCTION WORK .... D<br>SEX WORK .... E<br>OTHERS(SPECIFY) .... X<br>DON'T KNOW .... Y	

NO.	QUESTIONS	CODING CATEGORIES	SKIP
**Accessibility of secondary school to young SC/ST Girls**			
409	In your village, how difficult is it for girls like you to complete secondary school?	VERY DIFFICULT .... 1<br>SOMEWHAT DIFFICULT .... 2<br>NOT VERY DIFFICULT .... 3<br>DON'T KNOW 98	
410	Now I'm going to read a list of challenges and I want you to tell me how much they interfere with girls like you being able to complete their secondary education? For each item, please answer either: A lot, Somewhat, or Not so much		

		A LOT	SOMEWHAT	NOT SO MUCH	
A	Paying for fees, books, uniforms and other school-related costs	1	2	3	
B	Getting to and from school	1	2	3	
C	Fear of harassment or teasing from boys	1	2	3	
D	Lack of decent toilet facilities for girls at school	1	2	3	
E	Poor treatment of girls by teachers	1	2	3	

NO.	QUESTIONS	CODING CATEGORIES	YES	NO	SKIP
411	We know that there are sometimes problems in schools. Thinking now about your current school (or the last one you attended), which of the following descriptions would you say apply?<br>**(READ OUT THE LIST AND TICK ALL SHE SAYS APPLY.)**	A. NOT ENOUGH TEXTBOOKS	1	0	
		B. QUALITY OF TEACHERS IS POOR	1	0	
		C. CLASSROOMS ARE DIRTY	1	0	
		D. CLASSROOMS ARE CROWDED	1	0	
		E. NO SEPARATE TOILETS FOR GIRLS	1	0	
		F. TEACHER OFTEN ABSENT FROM CLASS	1	0	
		G. TEACHERS DRUNK	1	0	
		H. GIRLS ARE TREATED POORLY COMPARED TO BOYS	1	0	
		I. SEXUAL HARASSMENT OF GIRLS BY OTHER STUDENTS/	1	0	
		J. SEXUAL HARASSMENT OF GIRLS BY TEACHERS/STAFF	1	0	
		K. HARSH PHYSICAL PUNISHMENT BY TEACHERS	1	0	
		L. BULLYING BY OTHER STUDENTS	1	0	
	**Now I'm going to ask you some questions about your hopes for the future**				
412	How important is it to you that you complete secondary school?	VERY IMPORTANT .... 1<br>SOMEWHAT IMPORTANT .... 2<br>NOT AT ALL IMPORTANT .... 3<br>DON'T KNOW 98			

NO.	QUESTIONS	CODING CATEGORIES	SKIP
413	CHECK Q 330: CODE '5' OR '6' ☐ CIRCLED ↓	CODE '1','2','3','4' ☐ CIRCLED	→ 416
414	How important is it to you that you marry someday?	VERY IMPORTANT .... 1<br>SOMEWHAT IMPORTANT .... 2<br>NOT AT ALL IMPORTANT .... 3<br>DON'T KNOW 98	
415	How important is it to you that you marry before age 18?	VERY IMPORTANT .... 1<br>SOMEWHAT IMPORTANT .... 2<br>NOT AT ALL IMPORTANT .... 3<br>DON'T KNOW 98	
416	How important is it to you that you have steady employment when you are an adult?	VERY IMPORTANT .... 1<br>SOMEWHAT IMPORTANT .... 2<br>NOT AT ALL IMPORTANT .... 3<br>DON'T KNOW 98	
417	In your opinion, how would completing secondary school education benefit you and other girls in your community?<br>**DO NOT READ OUT THE RESPONSES**<br>**(MULTIPLE RESPONSE POSSIBLE)**<br>**(Probe: Can you think of any other benefits?**	BETTER JOBS .... A<br>GREATER RESPECT BY OTHERS .. B<br>BETTER HUSBAND .... C<br>DELAYED MARRIAGE .... D<br>HEALTHIER CHILDREN .... E<br>MORE CONFIDENCE/SELF ESTEEM F<br>OPENS OPTIONS FOR HIGHER EDUCATION . G<br>OTHER(SPECIFY) .... X<br>NO BENIFITS .... Y	
	**Now I'm going to ask you the same questions but I want you to tell me your parents views on these matters**		
418	How important is it to **your parents** that you transition into secondary school (enter 8th standard?)<br>**(Probe: is that very important, somewhat important, or not at all important to them)**	VERY IMPORTANT .... 1<br>SOMEWHAT IMPORTANT .... 2<br>NOT AT ALL IMPORTANT .... 3<br>DON'T KNOW 98	
419	How important is it to **your parents** that you complete secondary school (class 10)	VERY IMPORTANT .... 1<br>SOMEWHAT IMPORTANT .... 2<br>NOT AT ALL IMPORTANT .... 3<br>DON'T KNOW 98	
420	CHECK Q 330:<br>CODE '5' OR '6' ☐ CIRCLED ↓	CODE '1','2','3','4' ☐ CIRCLED	→ 423
421	How important is it to **your parents** that you get married some day?	VERY IMPORTANT .... 1<br>SOMEWHAT IMPORTANT .... 2<br>NOT AT ALL IMPORTANT .... 3<br>DON'T KNOW 98	
422	How important is it to them that you marry before age 18?	VERY IMPORTANT .... 1<br>SOMEWHAT IMPORTANT .... 2<br>NOT AT ALL IMPORTANT .... 3<br>DON'T KNOW 98	} 424

NO.	QUESTIONS	CODING CATEGORIES	SKIP
423	How important is it to your husband that you complete secondary school?	VERY IMPORTANT .... 1<br>SOMEWHAT IMPORTANT .... 2<br>NOT AT ALL IMPORTANT .... 3<br>NOT APPLICABLE .... 97<br>DON'T KNOW 98	
424	How important is it to **your parents** that you have steady employment when you are an adult?	VERY IMPORTANT .... 1<br>SOMEWHAT IMPORTANT .... 2<br>NOT AT ALL IMPORTANT .... 3<br>DON'T KNOW 98	
425	Now I'm going to read a set of statements, and for each one, I want you to tell me whether you Agree, Somewhat Agree, or Disagree.		

	STATEMENTS	AGREE	SOMEWHAT AGREE	DO NOT AGREE	DON'T KNOW	
A	It is more important to send a son to school than to send a daughter	1	2	3	98	
B	A wife should always obey her husband.	1	2	3	98	
C	Girls should be married as soon as they menstruate	1	2	3	98	
D	It is equally important to raise a daughter to be strong and independent as it is a son	1	2	3	98	
E1	Girls who are harassed or teased encourage it by the way they act	1	2	3	98	
E2	Girls who are harassed or teased encourage it by the way they dress	1	2	3	98	
F	Daughters should be sent to school only if they are not needed to help at home	1	2	3	98	
G	Girls should have the same rights and freedoms as boys.	1	2	3	98	
H	It is okay for a husband to beat his wife if she has done something wrong	1	2	3	98	
I	It is equally important to encourage daughters to get an education as it is with sons	1	2	3	98	
J	In general, educated girls become rebellious and don't make good wives	1	2	3	98	
K1	One can't expect boys not to tease girls	1	2	3	98	
K2	Best way to protect girls is to keep them at home	1	2	3	98	
L	Girls should have a say in who they marry	1	2	3	98	
M	If there is limited money to pay for tutoring, it should be spent first on sons	1	2	3	98	
N	Girls should not be expected to serve or wait on their brothers	1	2	3	98	

	BLOCK V: SOCIAL NORMS				
	**EMPIRICAL EXPECTATIONS**				
501	Now I would like to ask you about what is common in your village. For each statement, tell me whether you agree, somewhat agree or disagree.				
	**STATEMENTS**	**AGREE**	**SOMEWHAT AGREE**	**DO NOT AGREE**	**DON'T KNOW**
A	The majority of families in my village do not send their girls to secondary school	1	2	3	98
B	The majority of families in my village educate their sons longer than their daughters	1	2	3	98
C	The majority of families in my village get their daughters married before age 18	1	2	3	98
D	The majority of families in my village do not give girls equal freedom as boys	1	2	3	98
E	The majority of families in my village blame girls if they get teased or harassed.	1	2	3	98
	**NORMATIVE EXPECTATIONS**<br>**[What others think I should do (Agree, Somewhat agree, Disagree)]**				
502	Now I want you to think of those people whose opinions matter most to you. I'm going to read another list of statements and I want you to again tell me whether you fully agree with the statement, agree somewhat but not completely, or disagree with the statement. OK, here we go:				
	**STATEMENTS**	**AGREE**	**SOMEWHAT AGREE**	**DO NOT AGREE**	**DON'T KNOW**
A	The majority of people important to me think I should complete secondary school	1	2	3	98
B	The majority of people important to me think that it is less important for me to get an education than my brother	1	2	3	98
C	The majority of people important to me think I should get married before age 18<br>18	1	2	3	98
D	The majority of people important to me think I should not have as much freedom as my brothers	1	2	3	98
E	The majority of people important to me think that I should be blamed if I get teased or harassed.	1	2	3	98
	**CONSEQUENCES FOR DEPARTING FROM NORMS**				
503	If you complete your secondary education, how likely is it that the following consequences might occur? (Very likely, somewhat likely, not likely)				
		**VERY LIKELY**	**SOMEWHAT LIKELY**	**NOT LIKELY**	
A	You will be teased and harassed by local boys or men	1	2	3	

		VERY LIKELY	SOMEWHAT LIKELY	NOT LIKELY	
B	You may encounter more arguments/conflicts with your parents	1	2	3	
C	You may find it more difficult to get married	1	2	3	
D	You may be considered uppity and disobedient	1	2	3	
503a	CHECK Q 330:<br>CODE '5','6','98','99' CIRCLED ☐ ↓<br>CODE '1','2','3','4' CIRCLED ☐ →				BLOCK VI
504	If you do not get married by 18, how likely is it that the following consequences might occur (Very likely, somewhat likely, not likely).				
		VERY LIKELY	SOMEWHAT LIKELY	NOT LIKELY	
A	You may find it difficult to find a husband	1	2	3	
B	You may be blamed for higher marriage expenses	1	2	3	
C	You may disappoint your parents	1	2	3	
D	You may be dedicated as a Devadasi	1	2	3	

BLOCK VI: SENSE OF SELF ESTEEM, ASPIRATIONS and CONFIDENCE

Self esteem, aspirations and confidence

Now I am going to ask you some questions about how you feel about yourself and how confident you feel in different situations. There are no right or wrong answers to these questions and you do not need to answer any questions that you do not want to.

601	Ask how much the respondent agree or do not agree with the following statements					
	**STATEMENTS**	**AGREE**	**SOMEWHAT AGREE**	**DO NOT AGREE**	**DON'T KNOW/**	
A	I feel I am as important as other siblings of my family.	1	2	3	98	
B	If I work hard, I feel capable of achieving my goals	1	2	3	98	
C	I can express my ideas well in school	1	2	3	98	
D	I feel as intelligent as most other people in my age.	1	2	3	98	
E	I do not have hope for my future (R)	1	2	3	98	
F	I am optimistic that I will have a better life than my parents.	1	2	3	98	
G	On the whole, I am satisfied with myself	1	2	3	98	

SELF EFFICACY						
602	Ask how much the respondent agree or do not agree with the following statements					
	**STATEMENTS**	**AGREE**	**SOMEWHAT AGREE**	**DO NOT AGREE**	**DON'T KNOW**	
A	I feel able to talk to my parents about my hopes and aspirations	1	2	3	98	
B	I can stand up for my right to be treated with the same respect as my brother.	1	2	3	98	
C	I can express my views on marriage even if they differ from those of my parents	1	2	3	98	
D	I can ask my parents to support my completion of secondary education	1	2	3	98	
E	I feel willing and able to speak out in support of girls education	1	2	3	98	
F	I feel willing and able to speak out against child marriage	1	2	3	98	
G	I feel willing and able to speak out against eve teasing	1	2	3	98	
H	I feel able to seek help from others to achieve my goals	1	2	3	98	

INCREASED SOCIAL NETWORK

603 Do you belong to any of the following groups? And how often do you attend?**READ EACH OPTION AND RECORD WHETHER BELONGS TO THE GROUP AND THEN FOR EACH GROUP RESPNDED AS "YES" ASK HOW OFTEN THEY MEET IN THE GROUP AND RECORD THE RESPONSE.**

TYPE OF GROUPS	BELONGS TO GROUP?		HOW OFTEN DO YOU ATTEND?				
	YES	NO	ALMOST EVERY DAY	AT LEAST ONCE A WEEK	AT LEAST ONCE A MONTH	LESS OFTEN	NOT AT ALL
A. PARIVARTAN GROUP	1	0	1	2	3	4	5
B. SPORTS GROUP	1	0	1	2	3	4	5
C. STUDY GROUP	1	0	1	2	3	4	5
D. LIFE SKILLS EDUCATION	1	0	1	2	3	4	5
E. DANCING/SINGING/MUSIC GROUP	1	0	1	2	3	4	5
F. SAVINGS GROUP	1	0	1	2	3	4	5
G. BHAJANA GROUP	1	0	1	2	3	4	5
H. KISHORI GROUP	1	0	1	2	3	4	5

604	Have you ever taken part in a public discussion, campaign or action against child marriage?	YES .... 1<br>NO.. 0<br>DON'T KNOW.............................. 98	
605	Have you ever taken part in a public discussion, campaign or action in support of girl's education?	YES .... 1<br>NO.. 0<br>DON'T KNOW.............................. 98	

NO.	QUESTIONS	CODING CATEGORIES	SKIP
606	How many close friends do you have?<br><br>**CLOSE FRIEND** MEANS SOMEONE YOU FEEL YOU COULD TALK TO IN CONFIDENCE IF SOMETHING WAS TROUBLING YOU AND YOU HOLD A LOT OF TRUST IN THAT PERSON	NUMBER OF CLOSE FRIENDS [ ][ ]<br><br>(RECORD '00' IF NO CLOSE FRIENDS)	
607	Did you make new friends in the last year?	YES .... 1<br>NO...................................... 0<br>DON'T KNOW.............................. 98	
608	The last time you needed help or advice, did any of your friends help you?	YES .... 1<br>NO...................................... 0<br>DID NOT NEED ANY HELP.................... 94<br>DON'T KNOW.............................. 98	
609	If something was troubling you (e.g. academic performance, your own health issues, harassment etc), is there someone in your family you feel you could talk to about this?	YES .... 1<br>NO...................................... 0<br>DON'T KNOW.............................. 98	(NO, DON'T KNOW) 611
610	Which family member(s) do you feel most comfortable talking to?	NO ONE .... 0<br>MOTHER .... 1<br>FATHER .... 2<br>SISTER .... 3<br>BROTHER .... 4<br>GRAND PARENTS .... 5<br>OTHERS (SPECIFY)________________________..96	
611	If something were troubling you about school, who would you go to first for advice and support?	NO ONE (I'D KEEP IT TO MYSELF)............ 0<br>FRIEND .... 1<br>PARENT .... 2<br>SIBLING .... 3<br>OTHER RELATIVE .... 4<br>TEACHER .... 5<br>OTHER TRUSTED ADULT .... 6<br>PROJECT OUTREACH WORKER ..... 7<br>PARIVARTAN GROUP .... 8<br>OTHER (SPECIFY)_______________________....96	
612	If you experienced conflict with your parents, who if anyone, would you go to for advice and support?	NO ONE (I'D KEEP IT TO MYSELF)............ 0<br>FRIEND .... 1<br>SIBLING .... 2<br>OTHER RELATIVE .... 3<br>TEACHER .... 4<br>OTHER TRUSTED ADULT .... 5<br>PROJECT OUTREACH WORKER ...... 6<br>PARIVARTAN GROUP .... 7<br>OTHER (SPECIFY)_______________________....96	
	**MARRIAGE DISCUSSION AND FEELINGS ABOUT THE DISCUSSION**		
613	Have you ever heard your parents discussing your marriage in the last 12 months?	YES .... 1<br>NO...................................... 0<br>NOT APPLICABLE.......................... 97<br>DON'T KNOW.............................. 98	(NO, NOT APPLICABLE, DON'T KNOW) **701**

		NO ANSWER....99	

NO.	QUESTIONS	CODING CATEGORIES	SKIP
614	When your parents discussed about your marriage in the past 12 months, did they discuss this with you?	YES ....1<br>NO....0<br>DON'T KNOW....98	
615	How did you feel when you heard your parents discussing your marriage?<br><br>(DO NOT READ THE RESPONSE, PROBE FOR MORE)	HAPPY....A<br>EXCITED ....B<br>ANXIOUS ....C<br>STRESSED ....D<br>INTERNAL PAIN ....E<br>ANGRY ....F<br>DEPRESSED ....G<br>SAD ....H<br>CONFUSED ....I<br>FRUSTRATED ....J<br>EMBARRASSED ....K<br>SURPRISED ....L<br>SHOCKED ....M<br>SHY ....N<br>OTHER(SPECIFY) ....X<br>DON'T KNOW ....Y	

	BLOCK VII: AWARENESS OF GOVERNMENT SHEMES AND SUBSIDIES TO ENCOURAGE EDUCATION AND INVOLVMENT IN OTHER PROGRAM ACTIVITIES								
701	As you may be aware, the government has different kinds of programmes and schemes to help girls from poor families to attend school. I'm going to read out a list of programmes and I want you tell me if you or your family are currently benefiting from any of these schemes?								
**Sl. no.**	**Schemes/subsidies**	**Are you currently participating in this programme?**			**Why are you not participating in the scheme?**				
		**YES**	**NO**	**NOT APPLICABLE**	**NOT AWARE OF THE SCHEME**	**NOT ELIGIBLE**	**NOT REQUIRED**	**APPLIED BUT DID NOT GET**	
**(1)**	**(2)**	**(3)**	**(4)**	**(5)**	**(6)**	**(7)**	**(8)**	**(9)**	
A	Pre Metric Hostels	1	0	97	1	2	3	4	
B	Post Metric Hostels	1	0	97	1	2	3	4	
C	Incentive scholarships	1	0	97	1	2	3	4	
D	Scholarship for high school going girls	1	0	97	1	2	3	4	
E	Pre metric scholarships	1	0	97	1	2	3	4	
F	Pre metric scholarship for the children of parents who are in unclean occupation i.e. Scavenging, Tanning and Flayin	1	0	97	1	2	3	4	
G	State Post metric scholarships	1	0	97	1	2	3	4	
H	Prize money for meritorious students	1	0	97	1	2	3	4	
I	Book Bank Scheme	1	0	97	1	2	3	4	
J	Kishori Shakthi Yojana	1	0	97	1	2	3	4	
K	Rajiv Gandhi scheme for empowerment of Adolescent girls – SABALA	1	0	97	1	2	3	4	
L	Akshara Dasoha – Miday Meal program	1	0	97	1	2	3	4	
M	Free Text Books & Note Books	1	0	97	1	2	3	4	
N	Free school uniforms	1	0	97	1	2	3	4	
O	Free bi-cycle	1	0	97	1	2	3	4	
X	Any others (specify)	1	0	97	1	2	3	4	

NO.	QUESTIONS	CODING CATEGORIES	SKIP
702	Are you currently attending any tutorial classes?	YES.. 1<br>NO 0	→ 704 (NO)
703	Who sponsors the tutorial classes you attend?	SCHOOL .... 1<br>PRIVATE .... 2<br>PROJECT/NGO .... 3<br>OTHER(SPECIFY) 96	705
704	What is the primary reason you are not attending any tutorial classes?	NOT REQUIRED FOR ME .... 1<br>DO NOT HAVE TIME .... 2<br>COSTS TOO MUCH .... 3<br>PARENTS/GUARDIANS DENIED...................<br>SCHOOL DID NOT ALLOW .... 5<br>OTHER(SPECIFY)........................... 96<br>NOT APPLICABLE/NOT ELIGIBLE 97	
705	Have you ever attended Parivartan Group Sessions?	YES.. 1<br>NO .... 0<br>DON'T KNOW 98	707 (NO, DON'T KNOW)
706	Did you complete the entire Parivatan Programme?	YES.. 1<br>NO 0	
707	Have you ever attended any of the following services/trainings in your school? If not, what is the reason for not participating?		

	SERVICES/TRAININGS	EVER AVAILED ?		CURRENTLY AVAILING?		WHAT IS THE REASON FOR NOT AVAILING?					
		YES	NO	YES	NO	NOT ELIGIB LE	NOT REQUIRED	DID NOT GET	NOT AVAILABLE AT MY SCHOOL	NO ANSWER	
A	CAREER COUNSELLING	1	0	1	0	1	2	3	95	99	
B	LIVELIHOOD/VOCATIONAL TRAINING	1	0	1	0	1	2	3	95	99	
C	LIFE SKILL TRAINING	1	0	1	0	1	2	3	95	99	
D	Leadership training	1	0	1	0	1	2	3	95	99	

BLOCK VIII: KNOWLEDGE OF HIV AND AIDS, EVE TEASING AND MENTAL HEALTH

Now I'd like to ask you a few questions HIV/ADIS.

NO.	QUESTIONS	CODING CATEGORIES	SKIP
801	Have you ever heard/read about HIV, the virus that causes AIDS?	YES .... 1<br>NO 0	→ 808 (NO)
802	Do you know how HIV is transmitted?	YES .... 1<br>NO 0	→ 804 (NO)

NO.	QUESTIONS	CODING CATEGORIES	SKIP
803	Can you please tell me all the ways you know?<br><br>**(Do not read out the list, but after each response, ask if there are any other ways)**	HAVING SEX WITHOUT A CONDOM .... A<br>UNSAFE BLOOD TRANSFUSION .... B<br>UNSTERILE INJECTIONS .... C<br>TRANSMITTED FROM MOTHER TO CHILD DURING PREGNANCY .... D<br>SHARING PERSONAL ITEMS .... E<br>OTHER (SPECIFY) .... X<br>DON'T KNOW .... Y	
804	Is it possible for a healthy looking person to have HIV/AIDS?	YES .... 1<br>NO .... 0<br>DON'T KNOW 98	
805	Can HIV be transmitted by mosquito bites?	YES .... 1<br>NO .... 0<br>DON'T KNOW 98	
806	Can HIV be transmitted by sharing cups and plates?	YES .... 1<br>NO .... 0<br>DON'T KNOW 98	
807	Can HIV be transmitted by kissing?	YES .... 1<br>NO .... 0<br>DON'T KNOW 98	
	**EVE TEASING**		
	**Sometimes as girls bodies mature, they begin to attract unwanted attention from boys and men.**		
808	In the past month, have you heard about other girls in your village getting sexually harassed or teased somewhere in the village or on their way to school?	YES .... 1<br>NO .... 0<br>DON'T KNOW .... 98<br>NO ANSWER 99	
809	Last time, when did you witness a girl being sexually harassed or teased somewhere in the village or on the way to school?	NEVER .... 0<br>WITHIN THE LAST MONTH .... 1<br>LONGER THAN A MONTH BUT WITHIN THE LAST YEAR .... 2<br>LONGER THAN A YEAR 3	→ 812 (NEVER)
810	What was the age of the person(s) who you witnessed sexually harassing or teasing other girls somewhere in the village or on their way to school last time? Under 18, over 18, or both?	AGED UNDER 18 YEARS .... 1<br>AGED OVER 18 YEARS .... 2<br>BOTH 3	
811	Who were the people who you witnessed sexually harassing or teasing other girls somewhere in the village or on their way to school last time?	SC/ST BOYS/MEN .... 1<br>NON SC/ST BOYS/MEN .... 2<br>BOTH .... 3<br>DON'T KNOW 98	
812	In the past month, have you heard about girls being sexually harassed, groped or teased by someone at school?	YES .... 1<br>NO .... 0<br>DON'T KNOW .... 98<br>NO ANSWER 99	
813	When did you last time actually witness a girl being sexually harassed, groped or teased by someone at school?	NEVER .... 0<br>WITHIN THE LAST MONTH .... 1<br>LONGER THAN A MONTH BUT WITHIN THE LAST YEAR	→ 815 (NEVER)

		.... 2<br>LONGER THAN A YEAR 3	
**NO.**	**QUESTIONS**	**CODING CATEGORIES**	**SKIP**
814	Who were the people who you actually witnessed sexually harassing, groping or teasing girls at school last time?<br>Was this done by another student, a young person not in school, a teacher, or another adult?	ANOTHER STUDENT .... 1<br>YOUNG PERSON NOT IN SCHOOL .... 2<br>TEACHER .... 3<br>ANOTHER ADULT .... 4<br>DON'T KNOW 98	
	**MENTAL HEALTH**		
	**Emotional health or emotional well-being affects us all and we all have times when we feel down or stressed or frightened. Over the last two weeks, how often have you been bothered by any of the following problems?**		
815	Feeling down, depressed, or hopeless?	NOT AT ALL (0 DAYS) .... 0<br>SEVERAL DAYS(1 TO 7 DAYS) .... 1<br>MORE THAN HALF THE DAYS(MORE THAN 7 DAYS) . 2<br>NEARLY EVERY DAY(PAST TWO WEEKS OR 14 DAYS) 3	
816	Trouble falling or staying asleep, or sleeping too much?	NOT AT ALL (0 DAYS) .... 0<br>SEVERAL DAYS(1 TO 7 DAYS) .... 1<br>MORE THAN HALF THE DAYS(MORE THAN 7 DAYS) . 2<br>NEARLY EVERY DAY(PAST TWO WEEKS OR 14 DAYS) 3	
817	Feeling tired or having little energy?	NOT AT ALL (0 DAYS) .... 0<br>SEVERAL DAYS(1 TO 7 DAYS) .... 1<br>MORE THAN HALF THE DAYS(MORE THAN 7 DAYS) . 2<br>NEARLY EVERY DAY(PAST TWO WEEKS OR 14 DAYS) 3	
818	Feeling bad about your self?	NOT AT ALL (0 DAYS) .... 0<br>SEVERAL DAYS(1 TO 7 DAYS) .... 1<br>MORE THAN HALF THE DAYS(MORE THAN 7 DAYS) . 2<br>NEARLY EVERY DAY(PAST TWO WEEKS OR 14 DAYS) 3	
819	Trouble concentrating on things, such as doing household chores, engaging in conversations or playing?	NOT AT ALL (0 DAYS) .... 0<br>SEVERAL DAYS(1 TO 7 DAYS) .... 1<br>MORE THAN HALF THE DAYS(MORE THAN 7 DAYS) . 2<br>NEARLY EVERY DAY(PAST TWO WEEKS OR 14 DAYS) 3	

GO TO BLOCK – IX

BLANK PAGE FOR ANY OTHER INFORMATION

CONFIDENTIAL: *FOR RESEARCH PURPOSE ONLY*

Study Number: ☐☐☐☐

NEW SECTION TO BE COMPLETED ANONYMOUSLY USING PEN AND PAPER

	**BLOCK IX: SEXUAL BEHAVIOUR AND PREGNANCY**		
	The next section of the interview deals with romantic behaviours, sexual behaviours, pregnancy and childbearing. For some people, these topics are private matters and can be embarrassing to discuss. Therefore we have created a short paper survey that you can fill out in complete privacy. I will never know what answers you give. When you are finished, I will ask you to seal you answers in an envelope and add them to this bag with lots of other people's answers. If you have any questions, however, I will be more than happy to answer them. Tick (√) the correct answer wherever the box is given.		
**NO.**	**QUESTIONS**	**CODING CATEGORIES**	
901	As girls grow into women, certain changes happen to their bodies. One of these changes is that they start to have menstrual periods. Have you had your first menstrual period yet?	1.YES .... ☐<br>2.NO .... ☐	
902	How old were you when you first started menstruating	AGE IN COMPLETED YEARS .... ☐☐<br>DON'T KNOW 98	
903	Sometimes as girl's bodies mature, they begin to attract unwanted attention from boys and men. In the past 12 months, have you been sexually harassed or teased?	1.YES .... ☐<br>2.NO .... ☐	
904	How often have you been teased in the last 12 months?<br><br>(TICK ONLY ONE RESPONSE)	1. ONCE OR TWICE IN 12 MONTHS .... ☐<br>2. LESS THAN ONCE A MONTH .... ☐<br>3. MORE THAN ONCE A MONTH .... ☐<br>4. AT LEAST EVERY WEEK .... ☐<br>5. ALMOST EVERY DAY .... ☐	
	**Thinking now about the last 3 months only:**		
905	Have you been sexually teased or harassed at school in the last 3 months?	1.YES .... ☐<br>2.NO .... ☐	
906	Have you been sexually teased or harassed in the last 3 months on your way to school?	1.YES .... ☐<br>2.NO .... ☐	

NO.	QUESTIONS	CODING CATEGORIES	
907	Have you been sexually teased or harassed in the last 3 months somewhere else in the village?	1.YES .... ☐<br>2.NO .... ☐	
908	Are you currently married?	1.YES .... ☐<br>2.NO .... ☐	
909	Have you ever had a boyfriend/lover?	1.YES .... ☐<br>2.NO .... ☐	
910	Have you ever had sexual intercourse? By sexual intercourse, I mean a man putting his penis into a woman's vagina.	1.YES .... ☐<br>2.NO .... ☐	
911	How old were you when you first had sexual intercourse?	AGE IN COMPLETED YEARS .... ☐☐<br>DON'T KNOW .... 98<br>NO ANSWER 99	
912	Why did you have sex this first time?<br><br>(TICK ONLY ONE RESPONSE)	1. I GOT MARRIED .... ☐<br>2. I WAS DEDICATED AS DEVEDASI .... ☐<br>3. I WANTED TO HAVE SEX .... ☐<br>4. I WAS TRICKED INTO HAVING SEX .... ☐<br>5. I HAD SEX BECAUSE I NEEDED MONEY ..... ☐<br>6. I WAS PRESSURED INTO HAVE SEX .... ☐<br>7. I WAS PHYSICALLY FORCED TO HAVE SEX ... ☐<br>8. OTHERS (PLESE SPECIFY)____________ .... ☐	
913	Since your first sex, have you ever had intercourse in exchange for food, school fees, gifts or other items?<br><br>(TICK ONLY ONE RESPONSE)	1. NEVER .... ☐<br>2. ONCE .... ☐<br>3. A FEW TIMES .... ☐<br>4. MANY TIMES .... ☐	

NO.	QUESTIONS	CODING CATEGORIES	
914	Would you have chosen to have sex with this person if you knew before hand that you would not receive food, gifts or other items in return?<br><br>(TICK ONLY ONE RESPONSE)	1. NEVER ....<br>2. ONCE ....<br>3. A FEW TIMES ....<br>4. MANY TIMES	
915	Have you ever engaged in sex work or explicitly negotiated money in exchange for sex.<br><br>(TICK ONLY ONE RESPONSE)	1. NEVER ....<br>2. ONCE ....<br>3. A FEW TIMES ....<br>4. MANY TIMES	
916	Before the age of 15, did someone ever touch or fondle you in a sexual way when you did not want them to, but they did not try to force you to have sexual intercourse?<br><br>(TICK ONLY ONE RESPONSE)	1. NEVER ....<br>2. ONCE ....<br>3. A FEW TIMES ....<br>4. MANY TIMES	
917	Before you were 15, did someone ever try to have sexual intercourse with you when you did not want to, but sex did not happen?<br><br>(TICK ONLY ONE RESPONSE)	1. NEVER ....<br>2. ONCE ....<br>3. A FEW TIMES ....<br>4. MANY TIMES	
918	Before you were 15, did someone ever force you to have sexual intercourse with them?<br><br>(TICK ONLY ONE RESPONSE)	1. NEVER ....<br>2. ONCE ....<br>3. A FEW TIMES ....<br>4. MANY TIMES	
	**Now I would like to ask you about certain common health matters.**		
919	Have you ever been pregnant?	1.YES ....<br>2.NO	
920	How old were you when you first got pregnant?	WRITE AGE IN YEARS	

NO.	QUESTIONS	CODING CATEGORIES	
921	What was the outcome of your first pregnancy?	1. BABY BORN ALIVE ....<br>2. BABY STILL BORN (BORN DEAD AT THE END OF THE PREGNANCY ....<br>3. MISCARRIAGE (PREGNANCY LOST BEFORE BABY WAS BORN) ....<br>4. INDUCED LOSS OR ABORTION	
922	How many times in your life have you been pregnant?	NUMBER OF TIMES	
923	Over the last two weeks, how often have you been bothered by thoughts that you would be better off dead, or of hurting yourself in some way?	1. NOT AT ALL (0 DAYS) ....<br>2. SEVERAL DAYS(1 TO 7 DAYS) ....<br>3. MORE THAN HALF THE DAYS(MORE THAN 7 DAYS) ....<br>4. NEARLY EVERY DAY(PAST TWO WEEKS OR 14 DAYS)	

THANK YOU
